# Redescription of *Temnothorax antigoni* (Forel, 1911) and description of its new social parasite *Temnothorax curtisetosus* sp. n. from Turkey (Hymenoptera, Formicidae)

**DOI:** 10.3897/zookeys.523.6103

**Published:** 2015-09-28

**Authors:** Sebastian Salata, Lech Borowiec

**Affiliations:** 1 Department of Biodiversity and Evolutionary Taxonomy, University of Wrocław, Przybyszewskiego, 63/77, 51-148 Wrocław, Poland

**Keywords:** Mediterranean subregion, Crematogastrini, taxonomy, Turkey, Greece, *Temnothorax*

## Abstract

*Temnothorax antigoni* (Forel, 1911) is redescribed basing on a new material from southwestern Turkey (Antalya province), Lesbos and Rhodes (Greece, Aegean and Dodecanese islands). The gyne of this species is described for the first time. *Temnothorax curtisetosus*, a new species of social parasite collected in a nest of *Temnothorax antigoni*, is described. Colour photos of both taxa are given. A key to the worker caste of the eastern Mediterranean species belonging to both *Temnothorax recedens* and *Temnothorax muellerianus* groups are provided.

## Introduction

The genus *Temnothorax* Mayr, 1861 is one of the most speciose in the Myrmicinae subfamily. The most recent catalogue lists 380 valid species and 47 valid subspecies (Bolton 2015). Most species are distributed in northern hemisphere, mostly in temperate and warm temperate habitats, including taxa occurring in mountain habitats. More than a half of the described taxa are known from Europe and the Mediterranean basin (Borowiec 2014). However, museum collections suggest that many species remain undescribed. Originally, the genus *Temnothorax* included only taxa related to *Temnothorax recedens* (Nylander), which were characterized by an extremely deep mesonotal groove. Subsequently, Bolton (2003) synonymized several genera with *Temnothorax* and moved most of the species, placed originally in the genus *Leptothorax*, to this taxon. Social parasitism is often encountered in this group of ants and parasitic species were usually described in the separate genera. A recent phylogeny of the subfamily Myrmicinae, based on molecular data, showed that the parasitic taxa are nested within *Temnothorax* and cause non-monophyly of the genus. As a consequence, they were also synonymized with *Temnothorax* (Ward et al. 2015).

*Temnothorax antigoni* (Forel, 1911), a member of *Temnothorax recedens* group, was described from Western Turkey and has been known only from the type specimen until the present study. Heinze (1988) listed Turkish members of the tribe Leptothoracini and cited *Temnothorax antigoni* with a comment: [good species ?]. The junior author collected recently a nest samples of this rare species in Lesbos, Rhodes and in SW Turkey. The nest from Turkey contained specimens of a new socially parasitic ant belonging to the former *Chalepoxenus*. Below we redescribe *Temnothorax antigoni* (Forel), described gyne of this species for the first time, and describe the new socially parasitic species.

## Material and methods

Specimens were compared using standard methods of comparative morphology. Photos were taken using a Nikon SMZ 1500 stereomicroscope, Nikon D5200 photo camera and Helicon Focus software.

All given label data are in their original spelling; a vertical bar (|) separates data on different rows and double vertical bar (||) separates labels.

### Abbreviations of repositories

DBET Department of Biodiversity and Evolutionary Taxonomy, University of Wrocław, Poland;

MNHW Museum of Natural History, University of Wrocław, Poland;

NHMC Natural History Museum of Crete, Heraklion, Greece;

SSC Sebastian Salata collection.

### Measurand indices:

EL eye length; measured along the maximum diameter of eye;

EW eye width; measured along the maximum width of eye (diameter perpendicularly to EL);

HL head length; measured in straight line from mid-point of anterior clypeal margin to mid-point of occipital margin in full-face view;

HW head width; measured above the eyes in full-face view;

MH mesosoma height; measured from the upper edge of mesonotum to the lowest point of the mesopleural margin, in lateral view;

ML mesosoma length; measured as diagonal length from the anterior end of the neck shield to the posterior margin of the propodeal lobe;

PH petiole height; maximum height of petiole in lateral view;

PL petiole length; maximum length of petiole in lateral view;

PPH postpetiole height; maximum height of postpetiole in lateral view;

PPL postpetiole length; maximum length of postpetiole in lateral view;

PPW postpetiole width; maximum width of postpetiole in dorsal view;

PW petiole width; maximum width of petiole in dorsal view;

SDL spiracle to declivity length; minimum distance from the center of the propodeal spiracle to the propodeal declivity;

SL maximum straight-line length of the scape;

SPBA maximum distance between outer margins of spines measured at the base;

SPT maximum distance between outer margins of spines measured at the top;

PSL propodeal spine length; measured from the center of the propodeal spiracle to the top of the propodeal spine.

Example of measurements: 1.617 ± 0.135 (1.073–1.717) = The average measurement ± standard deviation (range of variation).

### Indices

EI eye index; EL/HL × 100;

HI head index: HW/HL × 100;

SI scape index: SL/HL × 100;

SPI propodeal spines index; PSL/HW × 100.

All lengths are in millimeters.

## Descriptions

### Temnothorax antigoni

Taxon classificationAnimaliaHymenopteraFormicidae

(Forel, 1911)

Leptothorax (Temnothorax) antigoni Forel, 1911: 333; Heinze 1988: 87; Kiran and Karaman 2012: 25.

#### Material examined.

Syntype worker photograph examined: *Temnothorax antigoni* | ☿type Forel | Coccarinali | p. Smyrne (Forel) || Typus || Sp. *Temnothorax antigoni* | Forel || Coll. Forel. || ANTWEB | CASENT | 0909060 (Available from: https://www.antweb.org/specimen/CASENT0909060, accessed 21 June 2015).

#### Other examined material.

Turkey, Antalya Prov.: 5 gynes, 6 workers from the single locality; Greece, Rhodes: 12 gynes, 177 workers from 5 localities; Greece, Lesbos: 3 gynes, 70 workers, 5 males from 5 localities (for detailed data of examined material see Suppl. material 1).

#### Redescription.

Worker (n=20). Measurements and indices: HL: 0.659 ± 0.04 (0.581-0.721); HW: 0.521 ± 0.032 (0.458-0.581); EL: 0.125 ± 0.09 (0.112-0.142); EW: 0.094 ± 0.005 (0.089-0.106); SL: 0.641 ± 0.039 (0.578-0.704); ML: 0.814 ± 0.062 (0.715-0.927); PSL: 0.139 ± 0.026 (0.078-0.179); SDL: 0.113 ± 0.024 (0.044-0.145); PL: 0.306 ± 0.028 (0.257-0.358); PPL: 0.188 ± 0.017 (0.156-0.218); PH: 0.186± 0.014 (0.162-0.212); PPH: 0.191± 0.016 (0.165-0.223); SPBA: 0.143 ± 0.02 (0.112-0.179); SPT: 0.149 ± 0.022 (0.112-0.19); PW: 0.146 ± 0.015 (0.123-0.168); PPW: 0.221 ± 0.025 (0.179-0.268); HI: 79.0 ± 1.6 (76.4-81.6); EI: 18.8 ± 0.8 (17.3-20.3); SPI: 27.2 ± 3.4 (19.9-32.7); SI: 97.2 ± 1.6 (93.8-99.7).

**Figures 1–2.**
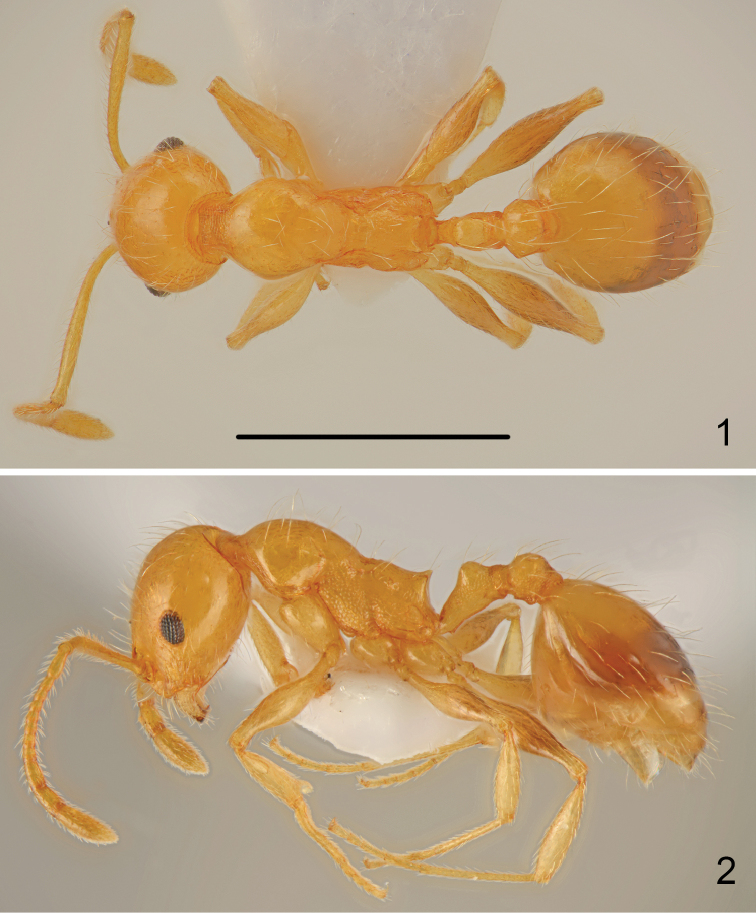
*Temnothorax antigoni* (Forel), worker **1** dorsal **2** lateral. Scale bar: 1 mm.

Whole body pale yellow, including antennae and legs, only first gastral tergite with pale, brown, regular transverse band apically (Figs 1, 2).

Head 1.2–1.3 times as long as wide, posterior margin of the head straight and laterally rounded in full-face view, gena almost parallel-sided (Fig. 7). Eyes small, 1.3 times as long as wide, gena 1.5 times as long as eye length, distance between line connecting hind margins of eyes to posterior margin of head 1.8 times as long as eye length. Anterior margin of clypeus regularly rounded, clypeal lines distinct, slightly divergent, reaching to line connecting anterior margin of eyes. Almost entire surface of head smooth and shiny, only gena with indistinct microreticulation. Clypeus, frons and top of head with numerous, long, erect hairs, the longest hair to 1.2 times longer than eye width, ventral surface of head with numerous long hairs, on the top of head hairs only slightly shorter. Antennal scape approximately as long as head, thin, in widest part only 1.8 times as wide as antennal base. Surface of scape smooth and shiny, covered with long, moderately dense, erect hairs. Funiculus 1.2 times as long as scape with three-segmented thin club, first segment twice longer than wide, second segment 1.3 times as long as wide, segments 3-5 approximately as long as wide, club very long, 0.75 times as long as segments 1-9 combined. Mesosoma elongate, 2.8 times as long as wide, with deep metanotal groove. Pronotum rounded on sides, regularly convex in profile, smooth and shiny, with 8-20 long, erect hairs. Promesonotal suture very fine but visible, mesonotum forms with pronotum regular arch, surface smooth and shiny with 4-8 long hairs. Mesopleura with regular granulate sculpture, metapleural suture distinct. Propodeum slightly convex in profile, surface with granulate sculpture but shiny, propodeal spines very short, triangular (Fig. 2), metapleura with granulate sculpture. Petiole elongate, 1.6 times as long as high, dorsal surface shallowly concave, petiolar lobe regularly rounded, ventral margin of petiole straight, carinate, with small, sharp denticle at the base. Petiolar lobe almost parallel-sided in dorsal view, then slightly converging to base. Petiolar lobe smooth and shiny with 4 long erect hairs, sides of petiole with granulate sculpture. Postpetiole globular in profile, from dorsal view slightly transverse with subangulate sides (Fig. 1), top of postpetiole smooth and shiny with 4-6 long, erect hair, sides with granulate sculpture. Gaster as long as mesosoma, surface smooth and shiny covered with numerous long, erect hairs (Fig. 2). Legs elongate, smooth and shiny, with sparse, semierect hairs, femora along underside with row of 3-4 long erect hairs. Hind tarsus 1.6 times as long as hind tibia.

#### Description.

Gyne (n=5). Measurements and indices: HL: 0.741 ± 0.012 (0.726-0.754); HW: 0.642 ± 0.015 (0.615-0.659); EL: 0.201 ± 0.08 (0.190-0.212); EW: 0.155 ± 0.06 (0.145-0.162); SL: 0.664 ± 0.023 (0.637-0.693); ML: 1.258 ± 0.025 (1.219-1.284); MH: 0.680 ± 0.044 (0.598-0.723); PSL: 0.199 ± 0.012 (0.184-0.218); SDL: 0.141 ± 0.013 (0.123-0.156); PL: 0.401 ± 0.025 (0.369-0.441); PPL: 0.237 ± 0.018 (0.212-0.257); PH: 0.277 ± 0.015 (0.257-0.302); PPH: 0.283 ± 0.007 (0.274-0.291); SPBA: 0.311 ± 0.006 (0.301-0.318); SPT: 0.281 ± 0.019 (0.251-0.302); PW: 0.200 ± 0.011 (0.190-0.223); PPW: 0.300 ± 0.017 (0.268-0.313); HI: 86.7 ± 1.5 (84.7-89.3); EI: 27.2 ± 1 (26.2-28.5); SPI: 31 ± 1.8 (28.4-34); SI: 89.6 ± 1.7 (87.7-91.9).

**Figures 3–4.**
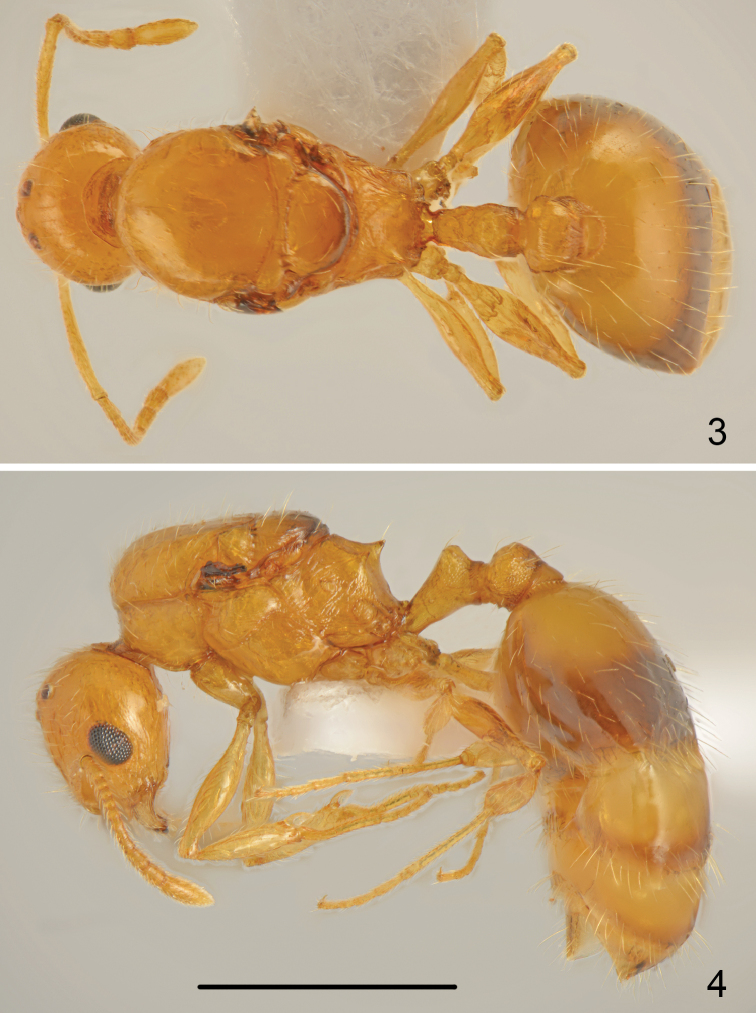
*Temnothorax antigoni* (Forel), gyne **3** dorsal **4** lateral. Scale bar: 1 mm.

Whole body pale yellow, including antennae and legs, only first gastral tergite with pale brown, regular transverse band apically and subsequent tergites with brownish posterior margin (Figs 3, 4).

Head 1.1 times as long as wide, posterior margin of head rounded in full-face view, gena almost parallel-sided (Fig. 8). Eyes large, 1.4 times as long as wide, gena 0.7 times as long as eye length, distance between line connecting hind margins of eyes to posterior margin of head 1.3 times as long as eye length. Anterior margin of clypeus regularly rounded, clypeal lines distinct, slightly divergent, reaching slightly behind line connecting anterior margin of eyes. Upper half of head smooth and shiny, frons on sides microreticulate but shiny, gena with rugose sculpture and along inner margin of eye run 2-3 thin carinae. Clypeus, frons and top of head with numerous, moderately long, erect hairs, the longest hairs slightly shorter than eye width, ventral surface of head with numerous moderately long hairs. Antennal scape 1.1 times as long as head, thin, in widest part only 1.6 times as wide as antennal base. Surface of scape smooth and shiny, covered with moderately long, moderately dense, more or less erect hairs. Funiculus 1.2 times as long as scape with three-segmented thin club, first segment twice as long as wide, second segment as long as wide, segments 3-5 elongate 1.3–1.4 times as long as wide, club long, approximately as long as segments 1-9 combined. Mesosoma 1.8 times as long as wide. Pronotum narrow, not visible from above, smooth and shiny. Scutum of mesonotum convex, smooth and shiny, covered with numerous moderately long, erect setae. Scutellum convex, smooth and shiny with view erect setae. Anepisternite with indistinct microreticularion, shiny, mesopleuron smooth and shiny. Propodeum short, surface with few transverse carinae, propodeal spines short, 1.1 times as long, acute, near apex with one long seta (Fig. 4), metapleura with distinct carinae. Petiole elongate, 1.5 times as long as high, dorsal side almost flat, petiolar lobe subangulate in profile, with short carina on sides, distinctly microreticulate, ventral margin of petiole straight, carinate, with small, sharp denticle at base. Petiolar lobe feebly rounded on sides in dorsal view, then distinctly converging to base. Petiolar lobe behind top microreticulate with two long setae. Postpetiole globular in profile, from dorsal view distinctly transverse, 1.3 times as wide as long, with carinate sides (Fig. 3), top of postpetiole microreticulate with several thin, longitudinal carinae and 7-9 long, erect setae, sides microreticulate with few short carinae. Gastral tergites smooth and shiny covered with numerous long, erect hairs (Fig. 4). Legs elongate, smooth and shiny, with moderately dense, semierect to erect hairs, femora along underside with row of 4-5 long erect hairs. Hind tarsus 1.7 times as long as hind tibia.

**Figures 5–6.**
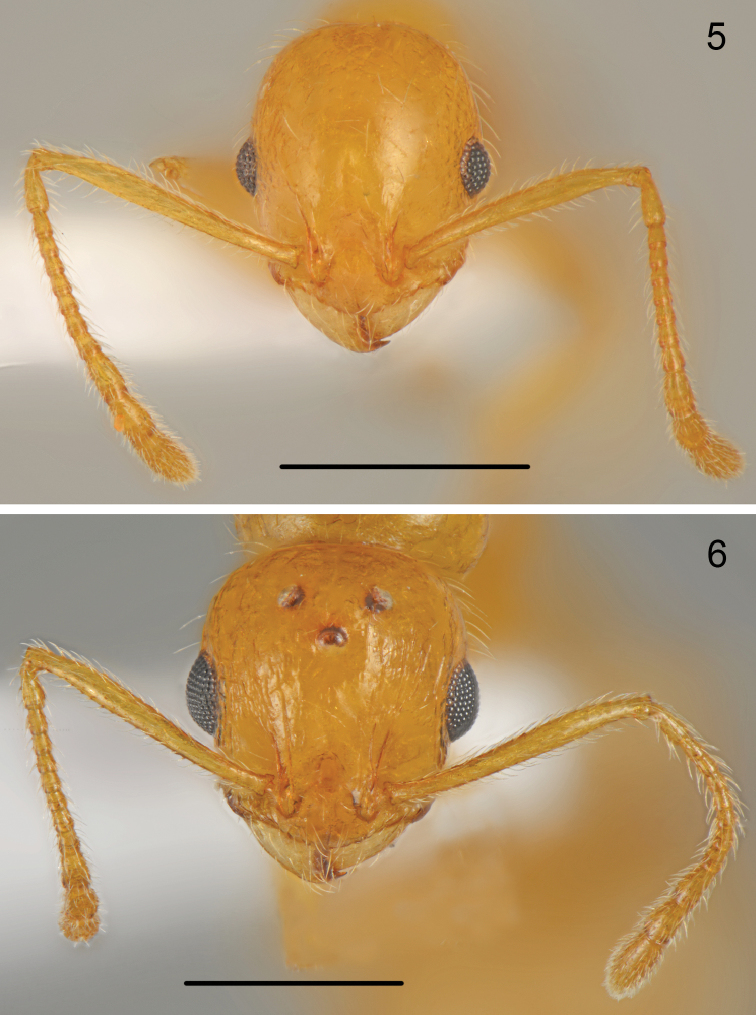
*Temnothorax antigoni* (Forel), head and antennae **5** worker **6** gyne. Scale bars: 0.5 mm.

**Figures 7–8.**
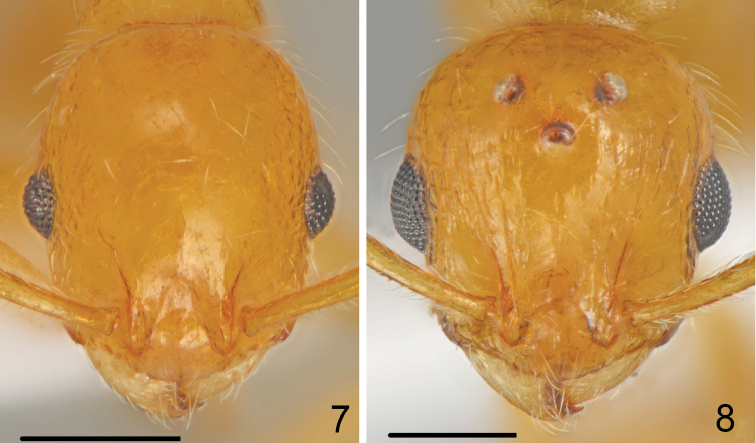
*Temnothorax antigoni* (Forel), head **7** worker **8** gyne. Scale bars: 0.25 mm.

#### Differential diagnosis.

*Temnothorax antigoni* is a species belonging to the former subgenus *Temnothorax* sensu stricto. The following related species occur in the eastern part of the Mediterranean: *Temnothorax finzii* (Menozzi) known from Italy, Macedonia and Turkey, *Temnothorax recedens* (Nylander) widespread in the Mediterranean area, *Temnothorax rogeri* Emery noted from Croatia, Montenegro, and Greece, and *Temnothorax solerii* (Menozzi) known from Greece (endemic to Karpathos island).

Workers of *Temnothorax finzii* distinctly differ by a very large eyes (EI > 24.8 in *Temnothorax finzii* vs EI < 20.3 in *Temnothorax antigoni*) and a longitudinal striation with rugosity covering entire lateral surface of the head while the head in *Temnothorax antigoni* is smooth and shiny. Another four species are very similar: *Temnothorax rogeri* differs in very long propodeal spine, at least twice as long as its width at base (in *Temnothorax antigoni* the spine is short, forms a denticle, not or only slightly longer than its width at base), *Temnothorax solerii* differs in entire body uniformly yellowish-brown to brown (in *Temnothorax antigoni* the body is uniformly pale yellow with darker transverse apical band on the first gastral tergite). At the first glance *Temnothorax antigoni* can be mistakenly determined as a pale variation of *Temnothorax recedens*. Workers of *Temnothorax recedens* are always bicoloured with head and gaster mostly dark and mesosoma usually with a darker spots on meso- and metapleura. Even pale workers of this species have always head and gaster gently darker than mesosoma with a pale basal spot on the first gastral tergite. In our collection we possess 17 gynes and 262 workers from 67 localities in Spain, Italy, Greece and Cyprus (see Suppl. material 1) and we have never found a specimens with colouration typical for *Temnothorax antigoni* (more than 230 examined specimens). In *Temnothorax antigoni* head and mesosoma are uniformly yellow, devoid of any darker discolourations and the gaster is mostly yellow with a darker transverse apical band on the first gastral tergite. This colouration is constant in all examined samples. The only observed variability was a degree of saturation of dark apical band on the first gastral tergite. Moreover, *Temnothorax antigoni* has average smaller eyes than *Temnothorax recedens* (EI: 18.8 ± 0.8 in *Temnothorax antigoni* vs 22.0 ± 1.6 in *Temnothorax recedens*).

Gynes are known only for *Temnothorax recedens* and *Temnothorax rogeri*. The gyne of *Temnothorax rogeri* distinctly differs in long propodeal spine, distinctly longer than width at base, head partly infuscate and gaster mostly brown with yellow spot at base of first tergite (in *Temnothorax antigoni* propodeal spine is triangular, as long as wide, body mostly uniformly yellow with darker transverse apical band on first gastral tergite and narrowly infuscate apical margin of subsequent tergites). The gyne of *Temnothorax recedens* differs in head and mesosoma usually bicoloured, with at least infuscate spot on meso- and metapleura, and mostly dark gaster (in *Temnothorax antigoni* the body is mostly uniformly yellow with darker transverse apical band on the first gastral tergite and narrowly infuscate apical margin of subsequent tergites).

#### Biological data.

In Turkey a nest of *Temnothorax antigoni* was found under a stone on a rocky side of a sandy path which runs through a pine forest. The locality is placed inside archeological site of the ancient Greek city Phaselis, close to the sea, only 6 m a.s.l. In the three Rhodes localities nests were found in rocks in mountain pine forest habitats at altitudes 522-598 m. Nests were located between schists of the volcanic rocks placed in the shade. In the five Lesbos localities nests were found in pine forest, oak forest and river valleys with platanus trees at altitudes 74-485 m. Nests were located under a moss overgrowing a large stones and between a schists of the volcanic rocks placed in the shade. A single workers were collected also on the surface of large stones or rocks. The following ant species were recorded in the same areas as *Temnothorax antigoni*:

Turkey, Antalya province, ancient Phaselis: *Aphaenogaster festae* Emery, *Aphaenogaster sporadis* Santschi, *Camponotus aegaeus* Emery, *Camponotus lateralis* (Olivier), *Camponotus rebeccae* Forel, *Camponotus samius* Forel, *Cardiocondyla bulgarica* Forel, *Crematogaster ionia* Forel, *Lasius neglectus* Van Loon, Boomsma & Andrasfalvy, *Lepisiota caucasica* (Santschi), *Lepisiota dolabellae* (Forel), *Lepisiota* sp., Messor cf. structor, *Pheidole koshewnikovi* Ruzsky, *Plagiolepis pallescens* sensu Radchenko, *Tapinoma* sp., and Tetramorium cf. semilaeve;

Greece, Rhodes, Attavyros loc. 2: *Aphaenogaster sporadis* Santschi, *Camponotus aegaeus* Emery, *Camponotus boghossiani* Forel, *Camponotus truncatus* (Spinola), *Crematogaster ionia* Forel, *Lepisiota melas* (Emery), *Pheidole koshewnikovi* Ruzsky, and *Temnothorax dessyi* (Menozzi);

Greece, Rhodes, Attavyros location 3: *Aphaenogaster festae* Emery, *Aphaenogaster sporadis* Santschi, *Camponotus boghossiani* Forel, *Camponotus kiesenwetteri* (Roger), *Camponotus lateralis* (Olivier), *Camponotus samius* Forel, *Crematogaster ionia* Forel, *Lepisiota melas* (Emery), *Temnothorax dessyi* (Menozzi), and *Plagiolepis taurica* Santschi;

Greece, Rhodes, road to Prof. Ilias location 2: *Aphaenogaster sporadis* Santschi, *Camponotus aegaeus* Emery, *Camponotus oertzeni* Forel, *Crematogaster ionia* Forel, and *Plagiolepis taurica* Santschi;

Greece, Lesbos, Ligona Valley: *Aphaenogaster balcanica* (Emery), *Aphaenogaster epirotes* (Emery), *Aphaenogaster lesbica* Forel, *Camponotus aegaeus* Emery, *Camponotus boghossiani* Forel, *Camponotus gestroi* Emery, *Camponotus lateralis* (Olivier), *Camponotus samius* Forel, *Camponotus truncatus* (Spinola), *Cataglyphis nodus* (Brullé), *Cataglyphis viaticoides* (André), *Crematogaster ionia* Forel, *Dolichoderes quadripunctatus* (Linnaeus), *Lasius alienus* (Förster), *Messor oertzeni* Forel, *Messor orientalis* (Emery), *Messor wasmanni* Krausse, *Monomorium monomorium* Bolton, *Pheidole pallidula* (Nylander), *Plagiolepis pallescens* sensu Radchenko, *Prenolepis nitens* (Mayr), *Temnothorax bulgaricus* (Forel), Temnothorax cf. parvulus, Tetramorium cf. caespitum, *Tetrarmorium diomedeum* Emery, and *Tetramorium punctatum* Santschi;

Greece, Lesbos, Antissa: *Aphaenogaster festae* Emery, *Camponotus lateralis* (Olivier), *Cataglyphis nodus* (Brullé), *Crematogaster ionia* Forel, *Crematogaster schmidti* (Mayr), *Dolichoderes quadripunctatus* (Linnaeus), *Lepisiota frauenfeldi* (Mayr), *Messor orientalis* (Emery), *Pheidole pallidula* (Nylander), *Temnothorax bulgaricus* (Forel), and *Trichomyrmex perplexus* (Radchenko);

Greece, Lesbos, 3 km N of Kalloni: *Camponotus lateralis* (Olivier), *Camponotus sanctus* Forel, *Crematogaster ionia* Forel, *Messor orientalis* (Emery), *Plagiolepis pallescens* sensu Radchenko, *Temnothorax bulgaricus* (Forel), Temnothorax cf. exilis, and *Temnothorax semiruber* (André);

Greece, Lesbos, M. Pythariou: *Aphaenogaster festae* Emery, *Camponotus lateralis* (Olivier), *Cataglyphis nodus* (Brullé), *Crematogaster ionia* Forel, *Lepisiota frauenfeldi* (Mayr), *Liometopum microcephalum* (Panzer), *Pheidole pallidula* (Nylander), *Temnothorax bulgaricus* (Forel), Tetramorium cf. chefketi, Tetramorium cf. semilaeve, *Tetramorium rhodium* Emery;

Greece, Lesbos, Ipsilometopo: *Aphaenogaster balcanica* (Emery), *Aphaenogaster epirotes* (Emery), *Aphaenogaster festae* Emery, *Camponotus boghossiani* Forel, *Camponotus kiesenwetteri* (Roger), *Camponotus lateralis* (Olivier), *Camponotus samius* Forel, *Camponotus sanctus* Forel, *Cataglyphis nodus* (Brullé), *Cataglyphis viaticoides* (André), *Crematogaster ionia* Forel, *Crematogaster lorteti* Forel, *Lasius alienus* (Förster) *Lepisiota frauenfeldi* (Mayr), *Messor oertzeni* Forel, *Messor orientalis* (Emery), *Monomorium monomorium* Bolton, Pheidole cf. pallidula, *Plagiolepis taurica* Santschi, *Prenolepis nitens* (Mayr), Temnothorax cf. affinis, Temnothorax cf. luteus, Temnothorax cf. tristis, *Temnothorax bulgaricus* Forel, Tetramorium cf. punctatum.

#### Distribution.

Described from Turkey: “Coccarinali près Smyrne” [now Izmir, Izmir province]. New locality in Turkey (ancient Phaselis) is placed in Antalya province approximately 370 km southeast from the type locality, three localities on Rhodes (Greece, Dodecanese) are placed 231-139 km southwest from the second locality in Turkey, and localities on Lesbos are placed 100-120 km northwest from the type locality (Fig. 16). Species new to Greek fauna.

A key to the worker caste of the East Mediterranean species belonging to the *Temnothorax recedens* group.

**Table:** 

1	Head rectangular, rugulose with longitudinal striation	***Temnothorax finzii***
–	Head oval, smooth and shiny	**2**
2	Whole body uniformly brown to pale brown, Greece: Karpathos Is	***Temnothorax solerii***
–	Body bicoloured, at least gaster with darker transverse apical band on the first gastral tergite	**3**
3	Propodeal spines very long, claw-shaped, more or less curved apically	***Temnothorax rogeri***
–	Propodeal spines short, never claw-shaped, pointed more or less upward	**4**
4	Head and masosoma uniformly pale yellow, gaster pale yellow with darker transverse apical band on the first gastral tergite, EI < 20.3	***Temnothorax antigoni***
–	Head and mesosoma usually bicoloured, with at least infuscate spot on meso- and metapleura, gaster dark with pale basal spot of first tergite, EI > 20.3	***Temnothorax recedens***

### Temnothorax curtisetosus

Taxon classificationAnimaliaHymenopteraFormicidae

Salata & Borowiec sp. n.

http://zoobank.org/CF1D977F-E8B0-45AE-B747-21F5590949C2

#### Etymology.

Named after the very short setae on mesosoma dorsum and gastral tergites.

#### Material examined.

Holotype worker (MNHW no. 1226): TURKEY, Antalya Prov. | ancient Phaselis | c. 6 m, 36.5262N/30.5455E | 29 VI 2010, L. Borowiec || Collection L. Borowiec | Formicidae | LBC-TR00059 || *Temnothorax* | *curtisetosus* sp. n. | in nest of Temnothorax antigoni | det. Salata & Borowiec; paratype worker: the same data as holotype (DBET).

#### Description.

Measurements: Workers (n = 2). HL: 0.715-0.737 (0.726); HW: 0.536-0.570 (0.553); EL: 0.178-0.184 (0.181); EW: 0.145-0.151 (0.148); SL: 0.575-0.603 (0.589); ML: 0.899-0.905 (0.902); PSL: 0.162-0.170 (0.166); PL: 0.296-0.330 (0.313); PPL: 0.212-0.235 (0.2235); PH: 0.279-0.279 (0.279); PPH: 0.223-0.246 (0.2345); SPBA: 0.201-0.190 (0.1955); SPT: 0.229-0.223 (0.226); PW: 0.212-0.235 (0.2235); PPW: 0.313-0.313 (0.313); HI: 75-77.3 (76.2); EI: 24.9-25 (24.95); SPI 28.4-31.7 (30.1); SI: 80.4-81.8 (81.1).

**Figures 9–10.**
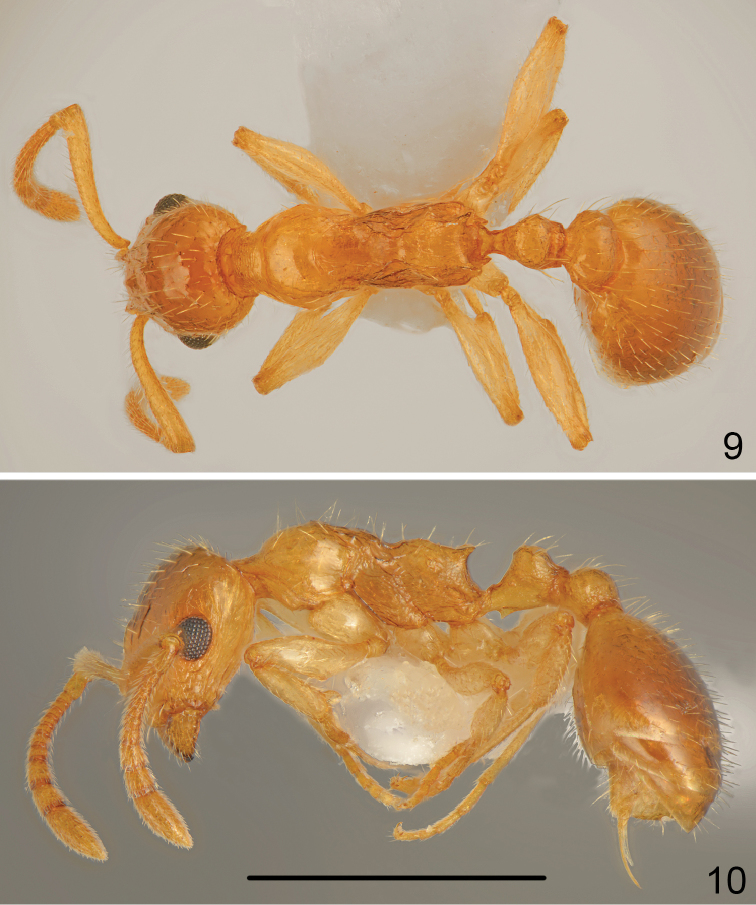
*Temnothorax curtisetosus* sp. n., worker **9** dorsal **10** lateral. Scale bar: 1 mm.

Head yellowish, in dorsal half slightly darker than in frontal parts and below eyes. Mesosoma, petiole, postpetiole, antennae and legs uniformly yellowish, first gastral tergite yellowish-brown with paler large patch at base, subsequent tergites yellowish-brown, sternites yellow (Figs 9, 10).

**Figure 11.**
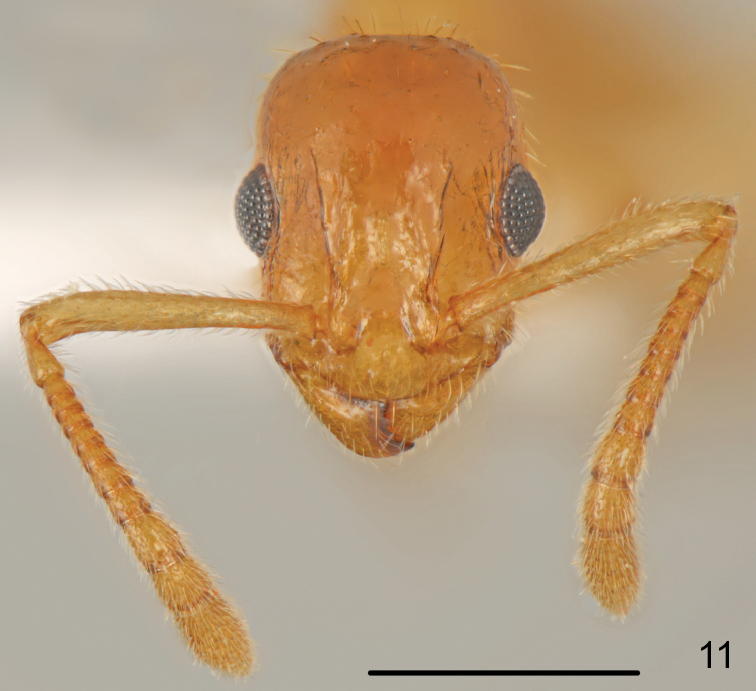
*Temnothorax curtisetosus* sp. n., worker head. Scale bar: 0.5 mm.

Head 1.4 times as long as wide, posterior margin of head straight and laterally rounded in full-face view, gena almost parallel-sided (Fig. 11). Eyes moderately large, 1.2 times as long as wide, gena 1.2 times as long as eye length, distance between line connecting hind margins of eyes to posterior margin of head 1.3 times as long as eye length. Anterior margin of clypeus regularly rounded, clypeal lines distinct, slightly divergent, reaching to the line connecting posterior margin of eyes. Almost whole surface of head smooth and shiny, only gena with rugose sculpture and along inner and outer margin of eye run 2–3 thin carinae. Clypeus, frons and top of the head with numerous, moderately long, erect hair, the longest hairs slightly shorter than eye width, ventral surface of head with numerous moderately long hairs. Antennal scape 0.8 times as long as head, thin, in widest part only 1.5 times as wide as antennal base. Surface of scape smooth and shiny, covered with moderately long, moderately dense, more or less erect hairs. Funiculus 1.3 times as long as scape with three-segmented thin club, first segment 1.8 times as long as wide, second segment as long as wide, segments 3–5 slightly transverse, club very long, only slightly shorter than segments 1–9 combined. Mesosoma elongate, 2.5 times as long as wide, with deep metanotal groove. Pronotum rounded on sides, regularly convex in profile, smooth and shiny, with 5–6 moderately long and few short, erect hairs. Promesonotal suture very fine but visible, mesonotum forms with pronotum regular arch, dorsal surface smooth and shiny with 4–8 moderately long hairs, sides with few longitudinal carinae. Mesopleura with indistinct microreticularion and few carinae. Propodeum distinctly convex in profile, surface with indistinct microreticulation but shiny, with 5–6 moderately long erect setae, propodeal spines short, 1.1 times as long as width at base, acute, near apex with one long seta (Fig. 10), metapleura with indistinct microreticularion and few carinae. Petiole short, 1.1 times as long as high, dorsal surface shallowly concave, petiolar lobe rounded, on sides with short carina, ventral margin of petiole straight, carinate, at base with moderately large, sharp denticle. In dorsal view petiolar lobe almost round, then distinctly converging to base. Petiolar lobe smooth and shiny with 4 long and two short erect hairs, sides of petiole microreticulate but shiny. Postpetiole globular in profile, from dorsal view distinctly transverse, 1.4 times as wide as long, with regularly rounded sides (Fig. 9), top of postpetiole smooth and shiny with 8–10 moderately long, erect hair, sides microreticulate with few short carinae. Gaster slightly shorter than mesosoma, surface smooth and shiny covered with numerous moderately long, erect hairs (Fig. 10). Legs elongate, smooth and shiny, with sparse, semierect to erect hairs, femora along underside with row of 3–4 long erect hairs. Hind tarsus 1.4 times as long as hind tibia.

#### Differential diagnosis.

*Temnothorax curtisetosus* sp. n. belongs to a monophyletic group of social parasites formerly classified as a separate genus *Chalepoxenus* Menozzi and recently synonymized with *Temnothorax* Mayr (Ward et al. 2015). The group comprises five species in Europe and the Mediterranean subregion, all are parasites of various *Temnothorax* species: *Temnothorax brunneus* (Cagniat) from Morocco, *Temnothorax kutteri* (Cagniat) from France: mainland, and Spain: mainland, *Temnothorax muellerianus* (Finzi) from Bulgaria, Croatia, Cyprus, France: mainland, Germany, Greece: Crete, Ionian Is., mainland, Sicily, Portugal, Serbia, Slovenia, Spain: mainland, Switzerland, Turkey and Ukraine, *Temnothorax inquilinus* Ward, Brady, Fisher & Schultz from Ukraine and *Temnothorax tramieri* (Cagniat) from Morocco.

**Figure 12–13.**
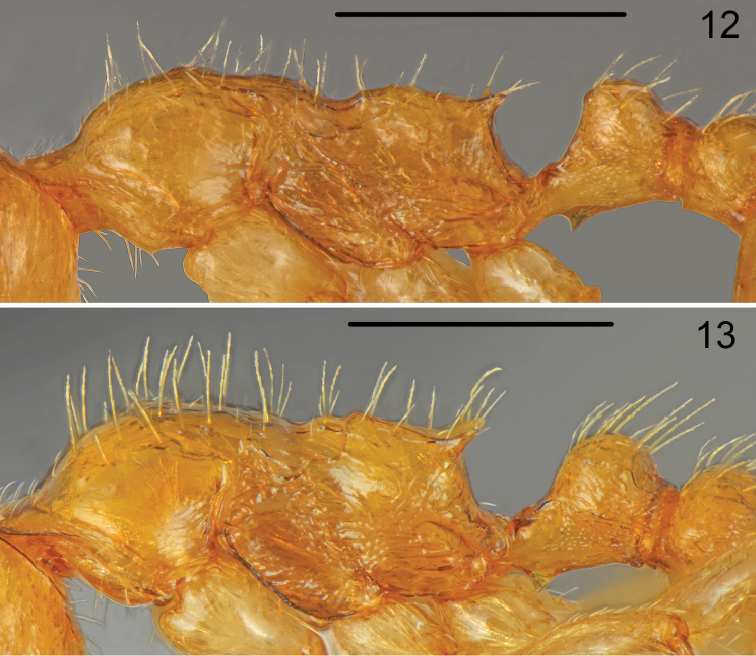
Worker mesosoma lateral **12** *Temnothorax curtisetosus* sp. n. **13** *Temnothorax muellerianus* (Finzi). Scale bars: 0.5 mm.

**Figure 14–15.**
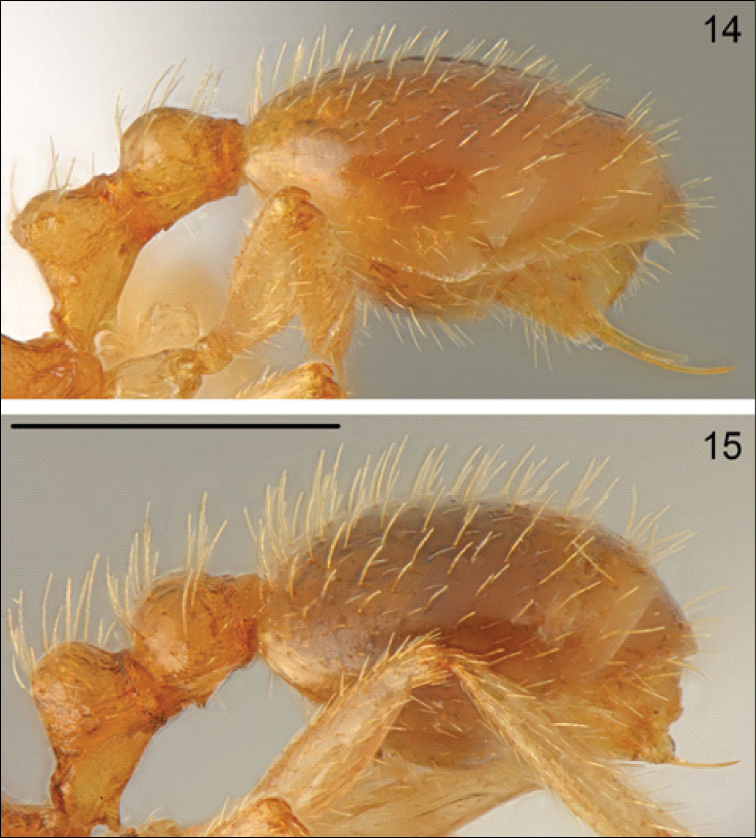
Worker gaster lateral **14** *Temnothorax curtisetosus* sp. n. **15** *Temnothorax muellerianus* (Finzi). Scale bar: 0.5 mm.

*Temnothorax curtisetosus* and *Temnothorax muellerianus* (Finzi) differ significantly from other members of this group in having tibiae covered with long, erect setae. *Temnothorax muellerianus* is the most widely distributed and the most variable species of this group (Buschinger et al. 1988). *Temnothorax curtisetosus* distinctly differs from *Temnothorax muellerianus* in very short setae on the mesosoma, petiole and postpetiole (Fig. 12 versus Fig. 13), and especially on gastral tergites (the total length of 10 setae combined on the first tergite is 741 µm in *Temnothorax curtisetosus* vs. 1111-1325 µm in *Temnothorax muellerianus* Figs 14–15). *Temnothorax curtisetosus* is smaller than most specimens of *Temnothorax muellerianus* and has shorter antennal scapes and higher SI index. At the first glance *Temnothorax curtisetosus* reminds a workers of *Temnothorax finzii*. Besides a clear differences in the biology of these species, *Temnothorax finzii* is a non-parasitic species inhabiting dry open habitats and nesting deep in the soil, usually under stone (Bračko et al. 2014), these species can be distinguished also in morphological features. *Temnothorax curtisetosus* differs from *Temnothorax finzii* by a weaker longitudinal striation covering only sides of the frons and its head is devoid of rugosity (in *Temnothorax finzii* whole head is covered by longitudinal striation with rugosity between it) and in presence of a dentiform plate on the ventral margin of the petiole, a character associated with a *Chalepoxenus* line. Moreover *Temnothorax curtisetosus* has also smaller propodeal spines (SPI < 31.7 vs SPI>40.8 in *Temnothorax finzii*).

**Figure 16.**
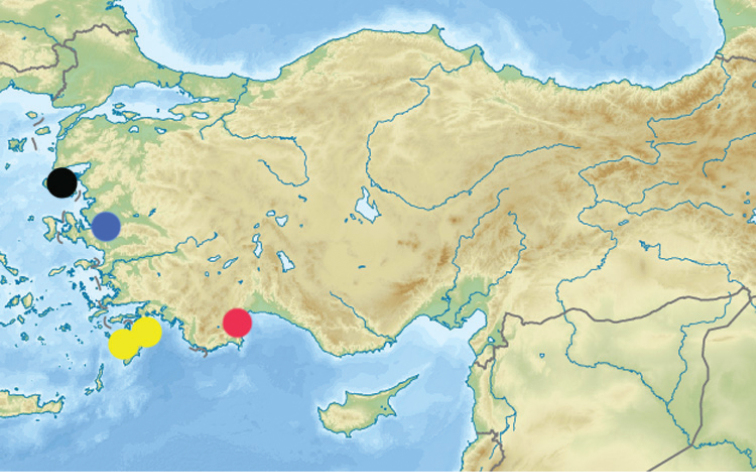
Distribution of *Temnothorax antigoni* (Forel), blue circle – locus typicus, red circle – new locality in Turkey (also locus typicus for *Temnothorax curtisetosus* sp. n.), yellow circles – new localities in Rhodes, black circle – new localities in Lesbos.

#### Distribution.

SW Turkey, Antalya Province.

#### Comments.

We found only two workers of *Temnothorax curtisetosus* in a nest of *Temnothorax antigoni*. The large number of gynes in relation to number of workers of the host species (5 gynes/6 workers) suggests that the nest was in the initial stage. Both specimens of the parasite have constant characters, especially very short dorsal setae. Very small specimens of *Temnothorax muellerianus* have dorsal setae proportionally 1.5 times longer than both specimens of *Temnothorax curtisetosus*. Kutter (1973) and Buschinger et al. (1988), basing on a large material from the entire Mediterranean basin, discussed variability and status of several taxa closely related to *Temnothorax muellerianus* but none of the samples studied by them were characterized by short dorsal setae. Although we have only two specimens of *Temnothorax curtisetosus*, the clear gap in the length of dorsal setae between these specimens and all examined samples of *Temnothorax muellerianus* (37 workers from 12 localities of 4 countries, see Suppl. material 1), a shorter propodeal spines and the analysis of variability within various populations of *Temnothorax muellerianus* discussed by Buschinger et al. (1988) convinced us to describe these two specimens as a species new for science.

A key to a worker caste of east Mediterranean species belonging to the *Temnothorax muellerianus* group is provided below.

**Table:** 

1	Tibiae bearing long, erect setae	**2**
–	Tibiae never with long, erect setae	***Temnothorax kutteri* group**
2	Setae on mesosoma and gaster short, the total length of 10 setae combined on the first gastral tergite less than 741 µm; propodeal spines short; SPI<31.7	***Temnothorax curtisetosus* sp. n.**
–	Setae on mesosoma and gaster long, the total length of 10 setae combined on the first gastral tergite more than 1100 µm; propodeal spines long; SPI>33.4	***Temnothorax muellerianus* (Finzi)**

**Table 1.** Comparative measurements of east Mediterranean species belonging to *Temnothorax muellerianus* and *Temnothorax recedens* groups.

WORKER							
	*Temnothorax recedens* group					*Temnothorax muellerianus* group	
	*Temnothorax antigoni* n=20	*Temnothorax finzii* n=2	*Temnothorax recedens* n=25	*Temnothorax rogeri* n=17	*Temnothorax solerii* n=20	*Temnothorax muellerianus* n=20	*Temnothorax curtisetosus* n=2
**HL**	0.659 ± 0.04 (0.581–0.721)	0.572–0.6	0.624 ± 0.05 (0.503–0.715)	0.675 ± 0.02 (0.636–0.726)	0.702 ± 0.034 (0.648–0.76)	0.816 ± 0.044 (0.715–0.888)	0.715–0.737 (0.726)
**HW**	0.521 ± 0.032 (0.458–0.581)	0.446–0.485	0.501 ± 0.05 (0.408–0.581)	0.552 ± 0.02 (0.518–0.598)	0.564 ± 0.032 (0.503–0.615)	0.615 ± 0.031 (0.536–0.668)	0.536–0.570 (0.553)
**EL**	0.125 ± 0.09 (0.112–0.142)	0.147–0.149	0.135 ± 0.016 (0.106–0.156)	0.152 ± 0.008 (0.14–0.168)	0.15 ± 0.009 (0.134–0.168)	0.209 ± 0.014 (0.179–0.235)	0.178–0.184 (0.181)
**EW**	0.094 ± 0.005 (0.089–0.106)	0.12–0.119	0.092 ± 0.015 (0.067–0.112)	0.105 ± 0.008 (0.095–0.123)	0.104 ± 0.006 (0.089–0.112)	0.168 ± 0.009 (0.156–0.179)	0.145–0.151 (0.148)
**SL**	0.641 ± 0.039 (0.578–0.704)	0.451–0.464	0.605 ± 0.06 (0.491–0.698)	0.677 ± 0.018 (0.648–0.709)	0.674 ± 0.035 (0.626–0.726)	0.637 ± 0.028 (0.57–0.693)	0.575–0.603 (0.589)
**ML**	0.814 ± 0.062 (0.715–0.927)	0.732–0.806	0.759 ± 0.095 (0.609–0.911)	0.831 ± 0.037 (0.782–0.893)	0.889 ± 0.053 (0.804–0.983)	0.992 ± 0.059 (0.893–1.106)	0.899–0.905 (0.902)
**PSL**	0.139 ± 0.026 (0.078–0.179)	0.188–0.1198	0.138 ± 0.027 (0.084–0.179)	0.216 ± 0.014 (0.19–0.243)	0.172 ± 0.016 (0.154–0.221)	0.237 ± 0.039 (0.179–0.363)	0.162–0.170 (0.166)
**SDL**	0.113 ± 0.024 (0.044–0.145)	0.106–0.104	0.126 ± 0.024 (0.087–0.162)	0.131 ± 0.009 (0.111–0.145)	0.131 ± 0.009 (0.123–0.151)	0.175 ± 0.01 (0.156–0.196)	0.123–0.123
**PL**	0.306 ± 0.028 (0.257–0.358)	/-0.291	0.284 ± 0.039 (0.212–0.346)	0.32 ± 0.026 (0.24–0.363)	0.337 ± 0.025 (0.296–0.374)	0.39 ± 0.03 (0.363–0.447)	0.296–0.330 (0.313)
**PPL**	0.188 ± 0.017 (0.156–0.218)	0.188-/	0.206 ± 0.035 (0.156–0.318)	0.232 ± 0.015 (0.201–0.257)	0.199 ± 0.016 (0.173–0.226)	0.303 ± 0.028 (0.279–0.346)	0.212–0.235 (0.224)
**PH**	0.186± 0.014 (0.162–0.212)	/-0.209	0.194± 0.026 (0.156–0.243)	0.213± 0.012 (0.201–0.24)	0.201± 0.016 (0.173–0.221)	0.283 ± 0.021 (0.268–0.335)	0.279–0.279 (0.279)
**PPH**	0.191± 0.016 (0.165–0.223)	0.205-/	0.19± 0.027 (0.145–0.243)	0.213± 0.014 (0.179–0.234)	0.205± 0.018 (0.173–0.235)	0.281 ± 0.023 (0.251–0.318)	0.223–0.246 (0.235)
**SPBA**	0.143 ± 0.02 (0.112–0.179)	0.143–0.149	0.137 ± 0.022 (0.089–0.19)	0.155 ± 0.011 (0.134–0.173)	0.163 ± 0.018 (0.134–0.19)	0.220 ± 0.019 (0.19–0.257)	0.201–0.190 (0.196)
**SPT**	0.149 ± 0.022 (0.112–0.19)	0.182–0.218	0.144 ± 0.023 (0.106–0.201)	0.154 ± 0.016 (0.19–0.246)	0.181 ± 0.02 (0.145–0.217)	0.265 ± 0.021 (0.223–0.302)	0.229–0.223 (0.226)
**PW**	0.146 ± 0.015 (0.123–0.168)	0.149–0.167	0.139 ± 0.018 (0.109–0.168)	0.154 ± 0.008 (0.145–0.168)	0.16 ± 0.013 (0.134–0.184)	0.244 ± 0.017 (0.212–0.279)	0.212–0.235 (0.224)
**PPW**	0.221 ± 0.025 (0.179–0.268)	0.22–0.253	0.209 ± 0.03 (0.156–0.257)	0.241 ± 0.012 (0.223–0.265)	0.246 ± 0.024 (0.212–0.279)	0.371 ± 0.037 (0.269–0.430)	0.313–0.313 (0.313)
**HI**	79.0 ± 1.6 (76.4–81.6)	78.0–80.8	80.3 ± 3.0 (71.9–85.9)	81.9 ± 4.2 (72.5–87.9)	80.3 ± 1.6 (76.3–82.8)	75.4 ± 1.8 (72.8–80.7)	75 -77.3 (76.2)
**EI**	18.8 ± 0.8 (17.3–20.3)	25.7–24.8	22.0 ± 1.6 (20.3.7–26.5)	22.5 ± 1.4 (20.0–24.8)	21.2 ± 1.3 (19.2–23.7)	25.6 ± 1 (22.9–27.7)	24.9–25 (24.95)
**SPI**	27.2 ± 3.4 (19.9–32.7)	42.2–40.8	27.2 ± 3.5 (20.6–34.4)	39.2 ± 3.0 (34.9–46.9)	30.4 ± 2.2 (27.3–37.3)	37.1 ± 2.0 (33.4–40.4)	28.4–31.7 (30.1)
**SI**	97.2 ± 1.6 (93.8–99.7)	78.8–77.3	96.9 ± 3.2 (92.3–108.1)	100.3 ± 2.8 (92.0–103.6)	96.0 ± 2.2 (92.2–101.6)	77.7 ± 2.2 (73.4–81.5)	80.4–81.8 (81.1)

## Supplementary Material

XML Treatment for Temnothorax antigoni

XML Treatment for Temnothorax curtisetosus

## Supplementary materials

Supplementary material 1 Table with specimens data Data type: specimens data This dataset is made available under the Open Database License (http://opendatacommons.org/licenses/odbl/1.0/). The Open Database License (ODbL) is a license agreement intended to allow users to freely share, modify, and use this Dataset while maintaining this same freedom for others, provided that the original source and author(s) are credited.Sebastian Salata, Lech Borowiec
